# Patients’ and caregivers’ experiences of using continuous glucose monitoring to support diabetes self-management: qualitative study

**DOI:** 10.1186/s12902-018-0239-1

**Published:** 2018-02-20

**Authors:** J. Lawton, M. Blackburn, J. Allen, F. Campbell, D. Elleri, L. Leelarathna, D. Rankin, M. Tauschmann, H. Thabit, R. Hovorka

**Affiliations:** 1 Usher Institute of Population Health Sciences and Informatics, University of Edinburgh, Edinburgh, UK; 2 Wellcome Trust-MRC Institute of Metabolic Science, University of Cambridge, Cambridge, UK; 3 Department of Paediatrics, University of Cambridge, Cambridge, UK; 4 Leeds Children’s Hospital, Leeds, UK; 5 Royal Hospital for Sick Children, Edinburgh, UK; 6 Manchester Diabetes Centre, Central Manchester University Hospitals NHS Foundation Trust, Manchester Academic Health Science Centre, Manchester, UK

**Keywords:** Continuous glucose monitoring, Type 1 diabetes, Qualitative, Patient experience, Caregiver experience

## Abstract

**Background:**

Continuous glucose monitoring (CGM) enables users to view real-time interstitial glucose readings and provides information on the direction and rate of change of blood glucose levels. Users can also access historical data to inform treatment decisions. While the clinical and psychological benefits of CGM are well established, little is known about how individuals use CGM to inform diabetes self-management. We explored participants’ experiences of using CGM in order to provide recommendations for supporting individuals to make optimal use of this technology.

**Methods:**

In-depth interviews (*n* = 24) with adults, adolescents and parents who had used CGM for ≥4 weeks; data were analysed thematically.

**Results:**

Participants found CGM an empowering tool because they could access blood glucose data effortlessly, and trend arrows enabled them to see whether blood glucose was rising or dropping and at what speed. This predicative information aided short-term lifestyle planning and enabled individuals to take action to prevent hypoglycaemia and hyperglycaemia. Having easy access to blood glucose data on a continuous basis also allowed participants to develop a better understanding of how insulin, activity and food impacted on blood glucose. This understanding was described as motivating individuals to make dietary changes and break cycles of over-treating hypoglycaemia and hyperglycaemia. Participants also described how historical CGM data provided a more nuanced picture of blood glucose control than was possible with blood glucose self-monitoring and, hence, better information to inform changes to background insulin doses and mealtime ratios. However, while participants expressed confidence making immediate adjustments to insulin and lifestyle to address impending hypoglycaemia and hypoglycaemia, most described needing and expecting health professionals to interpret historical CGM data and determine changes to background insulin doses and mealtime ratios. While alarms could reinforce a sense of hypoglycaemic safety, some individuals expressed ambivalent views, especially those who perceived alarms as signalling personal failure to achieve optimal glycaemic control.

**Conclusions:**

CGM can be an empowering and motivational tool which enables participants to fine-tune and optimize their blood glucose control. However, individuals may benefit from psycho-social education, training and/or technological support to make optimal use of CGM data and use alarms appropriately.

## Background

Continuous glucose monitoring (CGM) enables users to view real-time interstitial glucose readings and provides information on the direction and rate of change of blood glucose levels. Multiple alarms can be set to alert users if blood glucose either rises or falls (or is predicted to rise or fall) beyond predefined target ranges, and individuals are able to access historical data to inform diabetes self-management decisions. CGM is associated with reductions in glycated haemoglobin (HbA1c) levels in both adults and children, especially when used frequently [1–4]. CGM has also been shown to reduce hypoglycaemia and hyperglycaemia [2, 3, 5], severe hypoglycaemia [6]; and, improve treatment satisfaction [7–9] and quality of life outcomes [10, 11].

While the clinical and psychological benefits of CGM are well established, less is known about how individuals use CGM to make informed treatment decisions and why high levels of treatment satisfaction exist. Only limited qualitative research, focusing on user and/or caregiver experiences, has been conducted. This includes work exploring barriers to using, and reasons for discontinuing, CGM in adolescent [12] and adult groups [13]; adult users’ attitudes and characteristics which might help predict effective use of CGM [14]; and, how use of CGM may influence couple’s diabetes management and marital relationships [15]. More recently, Pickup et al. [16] used an open-ended survey question to explore user and caregiver experiences of CGM, including benefits and drawbacks encountered. While this is the most comprehensive study in terms of scope and sampling, Pickup et al.’s design did not permit user experiences to be explored in detail; hence, they recommended further, in-depth research be undertaken [16]. In line with this recommendation, we conducted in-depth interviews with individuals (adults, adolescents and parents) who made nonadjunctive use of CGM over ≥4 weeks in the initial training phase of a closed-loop study. The aim of this interview study was to understand and explore how participants used CGM to support diabetes self-management and what they considered the main benefits and drawbacks to be. Our objectives were to aid interpretation of findings from earlier CGM studies; and, provide recommendations for supporting individuals using CGM. An additional objective was to collect data to allow comparisons to be drawn with participants’ later experiences of using a closed-loop system as part of the trial; these data will be reported separately.

## Methods

### Participants and devices

Inclusion criteria for trial enrolment included a screening HbA1c ≥7.5% (58.5 mmol/mol) and ≤10% (86 mmol/mol) and a diabetes duration of at least 6 months [17]. Individuals were required to have used an insulin pump for at least 3 months, with good knowledge of insulin self-adjustment as judged by the investigator [17]. Individuals were ineligible for the trial if they had used CGM regularly in the previous three months.

Following trial recruitment, participants were trained to use the study insulin pump (MiniMed™ 640G pump, Medtronic, Northridge, CA, USA) and glucose sensor (Guardian™ Sensor 3, Medtronic by health care professionals who followed a common outline curriculum. Key areas covered in the training included: an insertion and initiation of sensor session, using the sensor menu of the insulin pump and sensor calibrations, use of software to analyse CGM data and use of CGM data to optimise treatment. Written guidelines for the operation and use of the CGM device were also provided in the form of the manufacturer’s user manual. Alarm settings on the CGM device were initially standardized but participants were allowed to adjust these during the study period. Parents/caregivers were unable to remote access their child’s CGM data.

Participants who took part in the interview study comprised: individuals aged ≥16 years; individuals aged 13–15 years and their parent(s)/caregiver(s); and, parents/caregivers of those aged ≤12 years. The decision to interview parents/caregivers of those aged ≤12 years and those aged 13–15 years was made because, in younger groups, parents take responsibility for most diabetes management tasks [18], while supporting and sharing responsibility with adolescents [19]. Interviewees were invited to take part in the qualitative study by members of the clinical team in the four participating UK sites (Cambridge, Manchester, Leeds, and Edinburgh). The clinical team informed these individuals that the qualitative research was being conducted by an independent research team and gave them reassurances of confidentiality. Recruitment and data collection continued until there was representation of different age groups in the final sample and data saturation had occurred; that is, until no new findings were identified in new data collected. The study received approval from the independent Cambridge East Research Ethics Committee (REC ref. 15/EE/0324). Participants aged ≥16 years and parents or guardians of participants aged < 16 years provided signed informed consent; written assent was obtained from minors before study-related activities.

### Qualitative study design

In-depth interviews were used as the method of data collection, as these afforded the flexibility needed for participants to discuss issues they perceived as salient, including those unforeseen at the study’s outset [20], while use of topic guides helped ensure the data collected remained relevant to the study aims and objectives. An inductive approach was used informed by general principles of Grounded Theory research [21]. This entailed simultaneous data collection and analysis, with findings from early interviews informing areas explored in later ones.

### Data collection and analysis

Interviews were conducted by MB at a time and location of participants’ choosing (mostly in their own homes) immediately before they moved into the main phase of the trial, at which point they had used CGM in real-life situations for a minimum of 4 weeks. Topic guides were developed based on literature reviews, input from clinical team members, and revised in light of emerging findings, in line with an inductive approach. Key areas explored included: previous experience of using CGM and self-monitoring of blood glucose (SMBG); understandings and expectations of CGM and impact of CGM (if any) on diabetes self-management; likes and dislikes of the technology; and views about information and training needed to support effective use of CGM. Patients aged 13–15 years old and their parents were interviewed separately. The interviews took place between July 2016 and May 2017. They typically lasted 1–2 h, were digitally recorded and transcribed in full for in-depth analysis.

Data were analysed by three experienced qualitative researchers (JL, MB and DR) using a thematic approach informed by the method of constant comparison; this entailed cross-comparison of all interviews to identify recurrent themes, before a coding framework was developed to capture these themes and contextual information needed to aid data interpretation. Nvivo, a qualitative software package, was used to facilitate data coding and retrieval and coded datasets were subjected to further analyses to allow more nuanced interpretations of the data to be developed.

## Results

The sample comprised 12 participants aged 16+ years, three participants aged 13–15 years and nine parents (see Table 1). A 100% opt-in was achieved. Eighteen interviewees (including six parents of child participants) described having had prior experiences of using CGM (e.g. to manage diabetes or as part of an earlier research study).

**Table 1:** Characteristics of study participants

Participants with type 1 diabetes (*n* = 15)		
Gender, female (*n*,%)		7(46.7)
Age at recruitment (years)		
	13–15	3
	16–20	2
	21–30	1
	31–40	6
	41–50	2
	51–60	
	60+	1
Occupation/education (n,%)		
	Professional	5(33.3)
	Semi-skilled	4(26.7)
	Retired	1(6.7)
	Higher education	2(13.3)
	Secondary school	3(20)
Self-reported diabetes duration (mean, SD, range - years)		
	≤12 years	4 ± 2.9 (2–9)
	13–17	9.25 ± 3.9 (4.5–13.75)
	18+	25 ± 11.1 (15–45)
Sensor use run-in (% over 4 weeks)		
	≤12 years	81.2 ± 13.8 (64–99)
	13–17	85.6 ± 10.8 (77–98)
	18+	89.9 ± 6.4 (77–97)
Parents of paediatric patients (*n* = 9)^a^		
Gender, female (*n*,%)		7(77.8)
Age at recruitment (years)		
	31–40	2
	41–50	5
	51–60	2
Occupation (n,%)		
	Professional	5(55.6)
	Semi-skilled	3(33.3)
	Unemployed/Full time Carer	1(11.1)

^a^This includes: parents who represented children aged ≤12 years (*n* = 5) and parents of children aged 13–15 (*n* = 4). In one instance, both parents of a child aged 13–15 participated in an interview

### Ease of access to continuous data

A key benefit of CGM, as all participants highlighted, was the ease with which they were able to access information about their blood glucose levels. Indeed, it was precisely because this process was so effortless that participants said they were much more aware of what their (or their child’s) blood glucose levels were throughout the day than when only SMBG was used:

“It’s so much easier with anything isn’t it, where you can just glance at it. It’s, you know, if to tell the time, rather than just glancing at your watch you had to sort of get something out, open it up, fiddle around with it, you wouldn’t worry so much about checking the time, would you?” (Parent 6)

Indeed, many participants drew a strong contrast between their experiences of using CGM and those of SMBG with Participant 3, like others, noting the limitations arising from the latter:

“it’s [SMBG] frustrating.. cause you don’t know what’s happening … it’s like walking around with a blindfold on. And you can walk into a room every now and then and take the blindfold off for 60 seconds. And then you have to put it back on.”

### Predicting and managing the future

As Participant 3 went on to elaborate, SMBG was of limited benefit not only because one could not instantly and effortlessly access one’s blood glucose levels, but also because it was not possible to establish “whether you’re going up or down, and how fast”. Indeed, a central benefit of CGM, as all participants observed, were the opportunities trend arrows presented to predict the future by virtue of being able to tell whether their blood glucose was rising or dropping and at what speed. This included Participant 1 who discussed how CGM data, when compared to SMBG results, enabled them to “make more informed decisions on what you’re going to do. And probably a lot sooner” because, as they explained, “I can see ok I’m actually going up. I’m going up really quickly. Or I’m coming down, I’m coming down fast, so I need to do something about it. As opposed to just a snapshot.”

As various individuals noted, CGM thus helped enable them to pre-empt and prevent hypo- and hyperglycaemia, and thereby achieve more stable blood glucose levels, because, “obviously you’re ready before it happens” (Parent 2). Specifically, participants discussed how the predictive information provided by CGM prompted proactive use of corrective insulin doses or consumption of carbohydrate to prevent their (or their child’s) blood glucose levels moving out of target ranges:

“if she [teenaged daughter] says: ‘oh I don’t feel quite right’, she can glance at it and think: ‘oh yeah I’m going high and I’ve got an arrow going straight up’. So she can then say: ‘oh yeah, actually I need to actually put a bolus in’. And equally if she’s got an arrow going straight down, she can say: ‘actually although I’m six, I’ve got an arrow going straight down. So I need something [to eat] within the next 20 minutes, half an hour, otherwise I’m gonna hypo’. So that’s good. You can almost stop things happening before they get to the critical point.” (Parent 6)

Some individuals also discussed how the predictive information provided by CGM enabled short-term lifestyle planning, whether this be, as Participant 2 described, by changing the timing of a meal or, in Participant 7’s case, reducing the length of a walk, to avoid hypo- or hyperglycaemia:

“It’s easier to do the stuff I wanna do, because I can read my pump. I can see: am I going up? Am I going down? Should I have lunch before I do this? Or can I do this before lunch kind of thing?... [using CGM] I can say: oh we’ll have lunch in half an hour. Or I can- I’ll just finish what I’m doing, or. So I think it’s giving me more personal freedom if you like.” (Participant 2)

“Yes, the arrow- well that’s good for going low. Em, it’s a good- great indicator to see that it’s- my sugars are actually- they’re dropping a little bit faster than I expected. They’re maybe dropping according to the graphs, but dropping a lot faster. Em, it means I can get, if I’m out for a walk with the dog, I can think: okay. Right I need to cut the walk short, because I’m going to go low otherwise.” (Participant 7)

While individuals described having received some instruction from health professionals on how to interpret and respond to the information provided by trend arrows, most emphasised that they found this information to be intuitive and easy to understand and as having prompted common-sense responses:

“they told me what the arrows mean… But I‘ve- I’ve kind of taken it upon myself to interpret that into three arrows and active insulin, get something quick, and quick-acting. Em so I’ve kind of used what I think is my best judgement on that.” (Participant 9)

### Understanding the impact of lifestyle and insulin on blood glucose levels

Participants also described how having easy access to CGM data on a continuous basis had enabled them to develop a much better understanding of how insulin, food and physical activity impacted on their blood glucose levels. As several individuals noted, such information had been an informative and motivational tool which had prompted positive changes to how they had approached and managed their diabetes. This included Participant 10 who described how they had broken a pattern of over-treating hypoglycaemia after ‘real time’ CGM data had provided evidence and reassurance that a more restrained approached was more efficacious:

“when I’d be treating a low, before I just thought I’ll just eat and eat. And, obviously the sensor showed me that when I did that I would just bounce way way back up, too high, because before, you know, I couldn’t physically see [this] on my pump with the arrows .. So now I know that I just- I can’t eat- I just don’t need to eat as much. I don’t need to panic as much.”

Participant 14, likewise, described adopting a more “patient” approach to their diabetes self-management in light of information provided by CGM, one which meant they were now less likely to over-correct for hyperglycaemia:

“So when I’ve used the pump to correct for it [high blood glucose] and I can see [from ‘real time’ CGM data], well actually I probably won’t need to, I can probably be a bit more patient and wait for it to catch up. And that’s quite helpful because that means I am less likely to have a hypo as a result of overcorrecting.”

In another pertinent example, Parent 2 noted how her 12 year old daughter has been motivated to make dietary changes after CGM data had alerted her to how consumption of high sugar foods, such as breakfast cereals, were causing her blood glucose to spike:

“she herself, off her own back, has been able to see, physically see on the line, where something has affected her blood sugar. Whereas before it will have affected it, and, you know, we treat it. But .. cause it’s a visual line she can see, that eating something’s gonna make her shoot up- it’s kind of- I think it’s struck a chord, in that she’s going: ‘well actually, that’s not really that good’. So she’s making conscious decisions in what she chooses to eat, without any kind of enforcement or- or guiding… changed the type of breakfast she has, so that she- she’s been having more smoothies rather than these high sugar, carby breakfasts.”

### Using the past to improve the future; retrospective analysis of data

Participants also discussed how the retrospective data provided by CGM enabled them to develop insight into their blood glucose control at times when they were much less likely to undertake SMBG, principally when they were asleep. In some cases, being able to examine graphs of night-time readings offered peace of mind that in target and stable blood glucose control was being achieved. In others, such as Participant 14, retrospective review of CGM data had alerted participants to unknown problems with their blood glucose control:

“And you can see what the history’s like and the trends and stuff. And I find that really, really helpful, because it’s like looking back over the period you’ve been asleep. And you can see what your blood sugar’s been doing... Sometimes you go to bed with a really normal blood sugar and you wake up and it’s normal. But over the night it’s just done this. And it’s like: ‘Wow’. Before I would have thought: ‘ah pretty good control really (laughs). But there’s something weird happening in the middle of the night.’” (Participant 14)

As participants also discussed, having access to graphs which captured historical data provided useful information which could be used to inform changes to basal rates and/or mealtime ratios:

“So you know, retrospectively you can look up the past couple of weeks and have a look at each day and you can see much better the patterns that come from your blood sugars and then you can adjust your insulin far more easily. … if you can see a pattern from the CGM charts I can change those instantly, the basal patterns and the ratios far more easily.. to get better stable control.” (Participant 3)

Indeed, while participants did note that it was possible to generate data for retrospective analysis using SMBG, because CGM captured data at five minute intervals, it also allowed a much more nuanced and informative picture to be generated than could be captured through periodic snapshots:

“Em, but since I’ve had the sensor, because I’ve got continuous points I’m getting a nice clean graph, rather than working off five or six points a day. So I’m able to make a lot better decision, rather than having to check my sugars every 30 minutes to try to get a nice trend pattern, I’m now getting a really good set of results that I can work off.” (Participant 7)

### Independent and dependent adjustments

However, while participants, including Participant 7, noted the value of the retrospective information provided by CGM, only a minority described having the confidence and ability to use this information to make independent adjustments to pump settings (basal rates) and/or meal time ratios. Indeed, in line with their earlier, pre-trial experiences of using insulin pumps (without CGM) where participants reported experiencing similar difficulties, the majority described both needing and expecting input and help from health professionals prior to changing basal rates and meal ratios. This was not only because participants questioned their own numeric skills and ability to analyse CGM data, but also because deferring to health professional expertise appeared, for many, to have been a habituated, taken-for-granted practice which pre-dated their use of CGM:.

“It’s quite- a quite complicated set up. So yes I have different basal rates, for different times. And we do change that. I don’t change it on my own...it’s not something I’d do without talking to some other clever girls…. only because I think it is so fundamental of my regime, that I would be worried about changing it without understanding 100% if it should be up or down. So it’s more insecurity I think, of my own knowledge about the basal.” (Participant 2)

“I see the consultant or the nurse often enough to do that with them…I wouldn’t feel that comfortable messing with- the insulin ratio- your insulin to carb ratios… because the ability to look at the data is less so than in the clinic, if that makes sense.” (Participant 4)

Some individuals, however, highlighted a need for education and training to make effective and independent use of historical CGM data while others, including Participant 7, pointed to the potential benefits of including pattern recognition software with future CGM devices:

“because I can see so many more variants in the line it makes me more determined to try and work out how to prevent, like monitor and check and find out why we have spikes and troughs … I’m at the stage- where at the moment I can do one change at a time. I know it just made me more determined to wanna be able to be in more control with her, working out what settings need changing, which is why I asked (names hospital) if they could give me that training.” (Parent 2)

“But it’s difficult to sort of detect the trends, which is where it’s nice in some software where it- it actually highlights stuff that it’s noticed.” (Participant 7)

### Tolerating and experiencing glitches and inaccuracies

In keeping with findings from previous studies [8, 13, 14, 16] participants reported various glitches and frustrations arising from using CGM. These included difficulties inserting and/or removing the device, finding a comfortable and discrete place on the body upon which to locate it, occasional loss of signal and challenges arising from needing to calibrate their devices at regular (12 hourly) intervals, especially when they did not lead routinized lives e.g. due to shift working. Participants, however, always tempered any criticisms with positive remarks and all emphasised that the clinical and psychological benefits of CGM outweighed any challenges encountered: “Calibration’s a pain but, you know, I’ve just got to do it…. And sort of it’s worth the effort” (Participant 4). Participants also indicated that that their tolerance of “technical hitches” (Participant 5) arose partly from their understanding that they were in a clinical trial, and their expectation that, over time, CGM technology would improve. In some cases, this expectation appeared to have resulted from earlier experiences of using CGM and observing developments in its accuracy and usability:

“and I’ve noticed.. as we’ve taken part in a lot of trials, these sensors are getting better.. the finger test and the sensor is becoming close and closer. I remember four, five years ago they were wide apart. And you thought, ‘well what’s the point, you know if it’s going to be so widely different.’ So we start seeing an improvement in the technology and how accurate the sensor’s becoming.” (Parent 5)

#### Alarms

In general, individuals pointed to clear clinical and psychological benefits to alarms alerting them to high/low blood glucose:

“It beeps when you’re going high. Having that, just that- that knowledge that you’ve got something looking out for you, just in case you do miss it, is- is so relieving, like ridiculously good… it’s just- just another level of freedom. You just- you know you’re safe… yeah, just added security.” (Participant 9)

Indeed, in one parent’s case, the existence of the alarm was believed to have saved her young child’s life:

“So, for instance a week ago, em [child’s name has] never had a hypo at 11 o’clock at night, never. I heard an alarm going off and thought: what the hell was that? He’d dropped to 2.2. I wasn’t due to test [child’s name] until 12 o’clock and I think it was about 11 o’clock. So I would have left him another hour, before I tested him. By then, he could have died or gone into a coma.” (Parent 7)

However, others noted how alarms could result in poor or interrupted sleep and/or unwelcomed distractions in the workplace or at school; with various children, including Parent 1’s daughter, reportedly switching alarms off in school due to concerns about drawing attention to themselves and distracting peers:

“The thing is she can’t really have the alarms at school cause the teacher is not very happy about her beeping. So, and if she does she just stops it so the kids are not looking at her because she’s beeping. So it doesn’t really have any kind of positive effect, the alarms during the day. So she would just switch them off. She doesn’t even check why is it beeping… Just because she don’t want them going off in school cause it‘ll draw attention to her.” (Parent 1)

Others described feeling that alarms ‘nagged’ them: “I suppose the worst of it would be the beeping… its beeping at you to tell you the sensor’s doing something, or it’s going too low, or something like that, so it nags” (Participant 3). This ‘nagging’ was described in particularly ambivalent ways by those, such as Participant 6, who suggested that she did not want to always lead a life dominated and dictated by her diabetes:

“I kind of love it and I hate it, cause I hate the fact that it shouted at me all the time. And they’re like [names health professional] ‘that’s what you want, so you can make sure you’re ok’. I’m like: ‘not at 3 o’clock in the morning, I don’t care.’” (Participant 6)

For similar reasons, Participant 10 also expressed ambivalence; in this individual’s case because the alarms acted as a tangible and difficult reminder not only that they had diabetes but also of their struggles to achieve optimal blood glucose control:

“It goes off a lot, it will vibrate and vibrate, and then this big alarm will go off… And it wakes me up. And it goes off in lessons. And it really frustrates me. And it’s like any diabetic will know if something annoys you about your diabetes, it’s more than being annoyed, it’s deep anger…it’s telling me I am high or it’s telling me I’m low.” (Participant 10)

#### Data lag

Others noted how, by virtue of the lag between CGM readings and actual blood glucose levels, they were sometimes exposed to information which was unhelpful:

“It alerts when I’m going low or I’m going high. So I do a sugar test: it says 13 the (CGM) reading says I’m 13.5 going up. So it’ll constantly beep… saying you’re 13.6, 13.7, you’re going up, you’re going up. And actually I’m going down. But it hasn’t caught up with it yet.” (Participant 13)

Indeed due to this lag and other occasional inaccuracies, some participants emphasised the importance of undertaking SMBG before addressing high/low blood glucose: “We don’t rely on it. You know if she’s [teenage daughter] having a hypo I’d still suggest that she tested” (Parent 6). Most participants, however, also described finding the lag relatively unproblematic because of how they actually made use of CGM data. For instance, it was noted that the small lag between actual blood glucose levels and CGM readings was unimportant when retrospective analysis of data was undertaken to spot patterns and trends which could inform changes to pump settings. In addition, when CGM data prompted participants to make more immediate changes to prevent high/low blood glucose, most noted how it was predictive information rather than actual blood glucose readings which they found most useful and informative:

“in a way… the arrows are more useful to you than the actual number because it’s not about what you are right now, is it. It’s about what’s gonna happen while you’re asleep, or while you’re going for your run... or whatever it is, like in a way that’s more helpful to you in terms of what’s happening next.” (Participant 6)

## Discussion and conclusions

This qualitative study has provided an in-depth understanding of participants’ experiences of, and views about, using CGM; and how, and why, CGM can be used to promote diabetes self-management. In doing so, we have offered a more detailed and nuanced perspective than is possible with quantitative/survey research. Specifically, our findings help explain why high levels of treatment satisfaction, reported in previous questionnaire studies [7–9], exist amongst CGM users. As we have shown, this is not only because CGM allows information about blood glucose to be accessed instantly and effortlessly, but also because trend arrows provide insightful information that enables diabetes to be managed in more proactive and effective ways than are possible with SMBG. Specifically, participants described using trend arrow information to pre-empt and prevent hyper- and hypoglycaemia by making proactive and appropriate use of carbohydrate consumption, corrective doses and short-term lifestyle planning. Such observations also help explain clinical research and trial findings that CGM can reduce hypo and hyperglycaemic excursions [2, 3, 5] and amplify and support findings of survey research undertaken with CGM users [22].

Participants also highlighted how CGM offered rich, informative data which enhanced knowledge of their blood glucose control at time points (e.g. night-time) when they were least likely to perform SMBG, and which could be used to adjust insulin basal rates and/or mealtime ratios to optimize or improve glycaemic control. However, while participants felt confident and able to make immediate, and what they saw as common-sense changes to lifestyle, food intake or insulin to address impending hypo- or hyperglycaemia, most described feeling much less confident and competent to undertake retrospective review of CGM data, spot patterns and trends and use these to inform independent adjustments to basal rates and mealtime ratios. While the former observation lends support to Bode and Battelino’s [23] suggestion that CGM usage remains largely intuitive, the latter raises important questions about whether the clinical benefits of CGM are always fully realised. Indeed, in keeping with observations and recommendations made by others [2, 11, 24] our findings point to a need for psycho-education and training amongst those using CGM to make optimal use of this technology. Specifically, as Ritholz et al. [14] have noted, we would recommend preparation and follow-up training about retrospective data use and analysis be given to individuals using CGM, a training need which has also been identified in other patient groups using flexible intensive insulin regimens [25, 26]. To address users’ education and training needs, staff training needs and workloads may also need to be taken into account [27]. The potential use of technologies, such as pattern recognition software, could also be considered [25]; indeed, such a recommendation was made by some of those who took part in this study. Another alternative may be to consider use of an individualised decision support system exploiting cloud-based technologies with or without health care professional input [28, 29].

Like others, we found that, while accounts of using CGM were overwhelmingly positive, participants encountered some difficulties and hassles using their devices [16]. These included calibration issues, equipment failure and problems inserting and/or removing the device [30]. While, in keeping with other studies, we found that disturbance caused by alarms could be a source of annoyance, especially to school-aged children [13, 30], our data reveal a richer and more complex picture. First, we have shown that, as well as causing frustration and disrupted sleep, alarms can also provide comfort and reassurance by alterting individuals to low (and high) blood glucose in a timely manner, thereby reinforcing a sense of hypoglycaemic safety [11]. Second, as our findings also suggest, some participants’ ambivalence about alarms appeared to arise from a more general dislike of having diabetes and an association made between alarming and what they saw as personal failure to achieve optimal blood glucose control. This is an issue which could be explored further in psychological work, to help determine whether some individuals, especially those with sub-optimal self-management behaviours and/or a high HbA1c, would benefit from psychosocial support prior to initiation of CGM to help maximise the benefits from alarms while preventing alarm fatigue [31]. However, on a more immediate and practical level, we would recommend that, as part of CGM training, individuals would benefit from instruction on how to switch alarms off and use different alarm profiles during different parts of the day. In addition, the snooze time of different alarms should be appropriately set to avoid repeated alarms.

While concerns about sensor inaccuracies and the lag between recorded and actual blood glucose levels have been highlighted by others [13], these were not found to be unduly problematic in the current study. While this may be due to improvements in CGM technology over time, we have also shown that participants found CGM data useful even if, because of the lag, they questioned the accuracy of readings. This was largely due to participants valuing predictive (trend arrow) information over actual readings, and also because retrospective review of data was not seen as being compromised by a small data lag. Some participants also emphasised the value of undertaking SMBG before taking action to address high/low blood glucose recorded by their sensors, a usage that may have been reinforced by the education and training they were given in the run up to the trial.

A key study strength is our use of an open-ended exploratory design which, as already indicated, offered a level and depth of insight not possible in clinical and survey research. An additional strength is the multicentre study design and the inclusion of a diverse age range of individuals in our sample, together with parents of those aged 15 years and under. This potentially means our findings have greater generalizability than those of other qualitative studies undertaken to date, although it should be noted that there is a skew in the sample towards those aged 31–40 years. Like others, [11, 14] our sample, which was recruited from a clinical trial, was heavily skewed towards well-educated/professional individuals, some of whom who had participated in earlier studies/trials of CGM. Consequently, such individuals may have been particularly motivated and interested in diabetes self-care and had an above average understanding of CGM. In addition, some participants’ earlier experiences of using CGM, and of seeing the technology improve over time, may have resulted in a form of ‘therapeutic optimism’ [32]. Specifically, participants may have hoped or believed that CGM technology will continue to improve, leading to overly positive and uncritical accounts. This may limit the generalizability of our findings; as may the fact that, in the currently study, only one particular CGM monitor was used and Polonsky et al.’s [11] observation that there are notable differences in usability, reliability and performance of CGM devices. In addition, we only focused on people’s experiences of using CGM for a limited number of weeks and it is possible that participants might have experienced fatigue had they used CGM for longer due to frustrations arising from alarms and CGM systems prompting action as soon as blood glucose moves out of predefined ranges [33, 34]. It should also be noted that, as we only interviewed people using insulin pumps, the findings may not be generalizable to those using injection regimens. For the aforementioned reasons, we would recommend further qualitative research be undertaken with more diverse socio-economic groups, recruited out with clinical trials, who use CGM for longer periods of time and/or who use multiple daily injection regimens.

## Abbreviations

CGM: Continuous glucose monitoring
DAFNE: Dose Adjustment for Normal Eating
HbA1c: Glycated haemoglobin
SMBG: Self-monitoring of blood glucose
